# Functional Diversity of Neurotrophin Actions on the Oculomotor System

**DOI:** 10.3390/ijms17122016

**Published:** 2016-12-01

**Authors:** Beatriz Benítez-Temiño, María A. Davis-López de Carrizosa, Sara Morcuende, Esperanza R. Matarredona, Rosa R. de la Cruz, Angel M. Pastor

**Affiliations:** Departamento de Fisiología, Facultad de Biología, Universidad de Sevilla, 41012 Sevilla, Spain; bbtmino@us.es (B.B.-T.); mayadavis@us.es (M.A.D.-L.d.C.); smorcuende@us.es (S.M.); matarredona@us.es (E.R.M.); rmrcruz@us.es (R.R.d.l.C.)

**Keywords:** trophic factors, nervous system, development, plasticity, axotomy

## Abstract

Neurotrophins play a principal role in neuronal survival and differentiation during development, but also in the maintenance of appropriate adult neuronal circuits and phenotypes. In the oculomotor system, we have demonstrated that neurotrophins are key regulators of developing and adult neuronal properties, but with peculiarities depending on each neurotrophin. For instance, the administration of NGF (nerve growth factor), BDNF (brain-derived neurotrophic factor) or NT-3 (neurotrophin-3) protects neonatal extraocular motoneurons from cell death after axotomy, but only NGF and BDNF prevent the downregulation in ChAT (choline acetyltransferase). In the adult, in vivo recordings of axotomized extraocular motoneurons have demonstrated that the delivery of NGF, BDNF or NT-3 recovers different components of the firing discharge activity of these cells, with some particularities in the case of NGF. All neurotrophins have also synaptotrophic activity, although to different degrees. Accordingly, neurotrophins can restore the axotomy-induced alterations acting selectively on different properties of the motoneuron. In this review, we summarize these evidences and discuss them in the context of other motor systems.

## 1. Introduction

During the last decades of the XXth century, increasing evidence pointed out the crucial role of neurotrophic factors, and in particular, neurotrophins, in the precise development of the nervous system. Target-derived neurotrophins regulate the extension of neuronal populations [1], and are involved in the acquisition of the adequate adult phenotype [2,3,4,5,6]. However, their functions are not restricted to the immature nervous system. Rather, they have been implicated in the regulation and maintenance of multiple adult functional properties under control conditions, such as membrane excitability [7,8,9], synaptic input stabilization [10,11] and synaptic plasticity [12,13,14], as well as in the maintenance of adult morphological phenotype [15]. In addition, neurotrophins participate in several mechanisms elicited in response to lesion, such as the synthesis and release of neurotrophins by activated glial cells, which may favor the axonal regeneration observed in injured motoneurons [16]. Moreover, exogenous administration of neurotrophins rescues at least some of the functions lost after injury in adult CNS neurons [11,17,18,19].

Several studies carried out in our group have demonstrated the relevance of neurotrophins and other trophic factors on the developing and adult oculomotor system. In the present review, we summarize these results and place them in the context of recent neurotrophin research.

## 2. Extraocular Motoneurons as the Experimental Model

**Figure 1:**
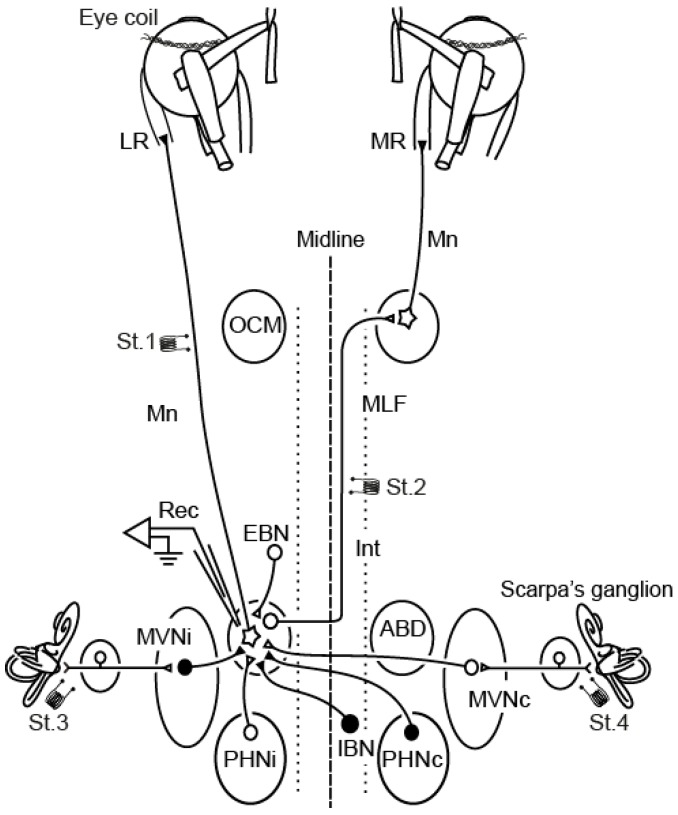
Diagram of the experimental design for chronic and acute recordings (Rec). The abducens nucleus (ABD) contains motoneurons (Mn) that innervate the lateral rectus muscle (LR) and internuclear neurons (Int) whose axons contact the contralateral medial rectus muscle (MR) innervating motoneurons of the oculomotor nucleus (OCM). Bipolar stimulating electrodes were implanted in the VIth nerve (St.1) and in the medial longitudinal fascicle (MLF; St.2); in some experiments, additional stimulation electrodes were implanted in the semicircular canals (St.3 and St.4). Inputs to ABD are illustrated: EBN and IBN, reticular excitatory and inhibitory burst neurons, respectively; PHN, prepositus hypoglossi nucleus; MVN, medial vestibular nucleus. The subscripts i and c indicate ipsilateral and contralateral, respectively, to the recorded ABD. Modified from [36].

The oculomotor system (and more precisely the abducens nucleus) has long been used as a model to study plasticity in the adult nervous system [20,21,22,23,24,25,26,27]. There are several points that make this motor system suitable for this line of research. First, its circuitry is very well established: eye movements are produced by three pairs of agonist/antagonist muscles that lead movements in the horizontal (lateral and medial rectus muscles), vertical (superior and inferior muscles) and oblique planes (inferior and superior oblique muscles). The activity of these muscles is controlled by motoneurons located in three different brainstem nuclei. Thus, the pontine abducens nucleus contains motoneurons that project through the ipsilateral VIth cranial nerve towards the lateral rectus muscle. Superior oblique muscle activity is under the control of contralateral trochlear motoneurons located at the ponto-mesencephalic junction, whose axons form the IVth cranial nerve. Finally, the mesencephalic oculomotor nucleus contains four different motoneuronal populations that control ipsilateral inferior and medial recti and inferior oblique muscle, as well as contralateral superior rectus muscle, through the IIIth cranial nerve [28]. Conjugate horizontal eye movements are elicited by a population of internuclear neurons located in the abducens nucleus [29]. Their axons cross the midline at its exit from the nucleus and project contralaterally through the medial longitudinal fascicle to innervate medial rectus motoneurons. Both abducens motor and internuclear neurons share the same threefold input system, that convey signals associated to either saccades (pontomedullary reticular formation), slow phases of the vestibular nystagmus (medial vestibular nucleus) or eye fixations (prepositus hypoglossi nucleus [28,30,31,32,33,34,35], see Figure 1).

Second, electrophysiological abducens neuron identification can be easily performed by antidromic activation from either the VIth nerve or the medial longitudinal fascicle for motor and internuclear neurons, respectively, and by their particular activity pattern in relation to eye movements. In fact, firing rate is closely related to eye position and velocity: both motoneurons and internuclear neurons exhibit a tonic firing rate that increases with more abducting ipsilateral eye fixations, and a phasic component of the firing rate that controls eye velocity [29,37]. Morphologically, specific identification of abducens motor and internuclear neurons by retrograde tracers injected in the lateral rectus muscle or in the contralateral oculomotor nucleus, respectively, is also feasible.

Third, both populations of neurons can be easily disconnected from their target, and in the case of motoneurons, it is also easy to provide them with exogenous neurotrophins from the periphery, without disturbing the integrity of the central nervous system.

Many experiments have revealed the strong dependence of abducens neurons on target-derived factors to survive during development, as well as to maintain the normal adult phenotype. For instance, in the neonatal period, the removal of extraocular muscles induces the death of their afferent motoneurons. Even more, medial rectus motoneuron retrograde death has a secondary effect on abducens internuclear neurons, which remain disconnected from their natural target (medial rectus motoneurons), and suffer the same process of cell death as a consequence of target loss [38]. As stated above, this retrograde programmed cell death has been described in other populations of neurons after target disconnection during development, leading to the statement of the Trophic Theory of Neural Connections, which postulates that developing neurons depend on the establishment of synaptic contacts with the appropriate target to be supplied with trophic factors that result essential for their survival [39,40].

Target dependence in the oculomotor system is not restricted to development. Instead, it is also crucial for the stability of mature neuron features. Experiments of toxin injection in the medial rectus muscle of adult animals, which led to motoneuron toxicity and death, were performed to produce the depletion of abducens internuclear neuron target cells. Target-deprived internuclear neurons suffered a process of soma shrinkage, dendritic retraction, and the loss of afferent contacts that, finally, produced changes in their normal firing pattern related to eye velocity and position [20,21].

Axotomy implies not only target disconnection, but also a direct damage to neuron integrity. However, the axotomy of both abducens populations produce similar results to those obtained after target depletion: loss of afferences and a reduction in the neuronal signals that codify eye position and velocity. In particular, firing modulation during eye fixations, spontaneous eye movements and the vestibulo-ocular reflex are clearly reduced [22,23,24]. All changes return to normality when neurons are able to reinnervate either their natural or even a different target. Thus, axotomized abducens motoneurons recovered their normal properties by three months after axotomy, probably due to muscle reinnervation [23]. Abducens internuclear neurons also established new contacts in the oculomotor nucleus after target depletion, which allowed the recovery of firing properties [20,21].

To fully demonstrate that the alterations that follow abducens internuclear neuron axotomy were due to target disconnection and the absence of target-derived signals, axotomized abducens internuclear neurons were provided with a different source of trophic factors, i.e., an implant of embryonic cerebellar primordia at the lesion site [25]. Abducens internuclear axons grew into the graft and established synaptic contacts with implanted cells, without affecting synaptic terminal morphology [22]. These and other experiments in which stem cells were implanted instead of cerebellar primordia [26] demonstrated that the establishment of new synaptic contacts, even with anomalous targets, restores afferent innervation pattern on abducens internuclear neurons [27], and thus, the normal firing pattern [41].

Summing up, these results indicate that all these alterations are induced by the lack of retrograde trophic support, rather than by axon damage. Thus, the new exogenous target would provide neurons with trophic molecules, restoring the retrograde flux of information, and therefore allowing the maintenance of afferent inputs and the electrophysiological properties. Our group has dedicated several years to understand which are the molecules implicated and how they control abducens neuron properties. The following sections explain these results in detail.

## 3. Extraocular Motoneurons Express the TrkA, TrkB and TrkC Receptors

Neurotrophins exert their function by binding to their high affinity receptors, the Trk subfamily of tyrosine protein kinases, which include the products of the trkA, trkB and trkC proto-oncogenes [42]. The relation of neurotrophins and their receptors is highly specific. Thus, nerve growth factor (NGF) activates TrkA [43,44], brain-derived neurotrophic factor (BDNF) [45] and neurotrophin-4/5 (NT-4/5) [46] binds to TrkB; and neurotrophin-3 (NT-3) [47] reacts with TrkC, although it can also bind TrkA and TrkB with lesser affinity [45,47,48].

**Figure 2:**
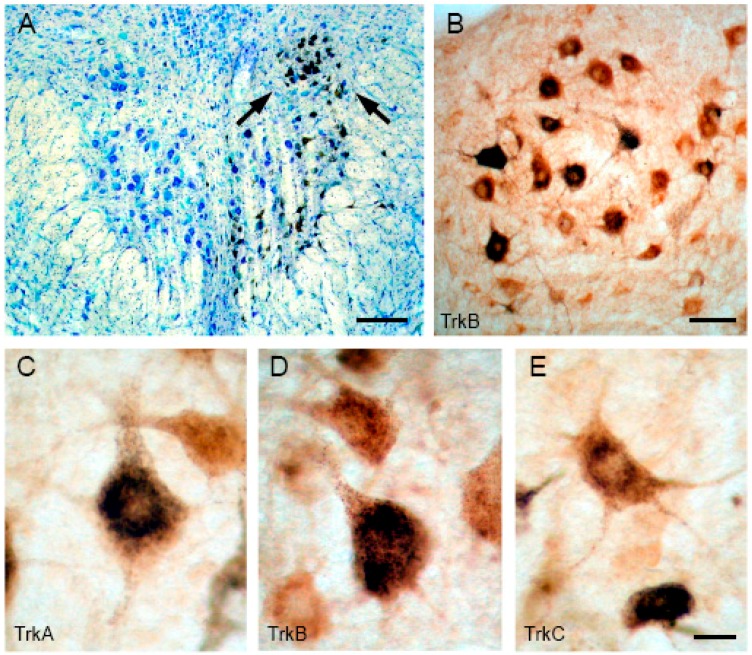
Trk staining of medial rectus motoneurons: (**A**) low-magnification image showing a coronal section through the oculomotor nucleus counterstained with Toluidin blue. HRP (horseradish peroxidase)-labeled cells between arrows at the dorsolateral column of the oculomotor nucleus correspond to medial rectus motoneurons; (**B**) combination of HRP retrograde labeling of medial rectus motoneurons and TrkB immunostaining in the oculomotor nucleus. Note the high proportion of double-stained cells; and (**C**–**E**) high-magnification pictures of HRP-identified motoneurons in sections immunostained against TrkA, TrkB and TrkC. Calibration bars: 250 µm for (**A**); 100 µm for (**B**); and 20 µm for (**C**–**E**). Modified from [49].

The alterations described in abducens neuron properties when deprived of retrograde trophic support could well be mediated by a deficit in neurotrophins. For this reason, as a first step in elucidating the role of neurotrophins in the plasticity of the oculomotor system, we characterized the distribution of Trk receptors in identified populations (i.e., motoneurons and internuclear neurons) of the extraocular motor nuclei of adult cats [49]. These experiments demonstrated the presence of the three different receptors in all populations of oculomotor cells, but also in preoculomotor regions, such as the vestibular and the prepositus hypoglossi nuclei. Figure 2 illustrates retrogradely-identified medial rectus motoneurons expressing TrkA, TrkB or TrkC.

The presence of TrkB had been demonstrated previously in the cat abducens nucleus [50] and chick oculomotor and trochlear motoneurons [51]. Likewise, the presence of TrkC had also been reported in the rat trochlear nucleus [52]. However, a full study of the expression of the three receptors in the three oculomotor nuclei with proper discrimination between neuronal populations was lacking.

Our results showed that almost all motoneurons were positive for the three receptors, indicating that most cells must co-express two or more neurotrophin receptors. In addition, other neurons present in the oculomotor nucleus were immunopositive against Trk antibodies. The same results were obtained for trochlear and abducens motoneurons and internuclear neurons. Co-expression of more than one Trk receptor is not exclusive of extraocular motoneurons. Thus, the giant cells of the pontine reticular formation in the adult cat [50], embryonic rat trigeminal ganglion [53], and neurons of the trigeminal system [54], ventral cochlear nucleus [55], visual cortex [56], dorsal root ganglion [57] and spinal and facial motoneurons of the adult rat also express two or the three neurotrophin receptors [58,59,60].

The finding that extraocular motoneurons of the abducens nucleus contain two or three Trk receptors indicated that these cells might be responsive to more than one neurotrophin. The actions of these growth factors during adulthood are different from those described during development, as mature neurons do not depend on neurotrophins for survival [15,21,48,61]. The co-expression of neurotrophin receptors in adult neurons might be associated to a high dependence on neurotrophin signaling for the maintenance of adult neuron functional and structural properties, as explained above. The possibility exists for neurotrophin receptors to regulate independent cellular functions, or to act in concert with each other regulating multiple aspects of the neuronal physiology during the normal operation of these oculomotor neurons, as will be discussed later.

Although all receptors were present in the different extraocular motoneuron populations, TrkB expression was prevalent. This receptor is also predominant in other motor systems such as spinal motoneurons [58,59]. The presence of this receptor in motoneurons suggests that they are responsive to BDNF, and, therefore, that they likely receive this factor from a natural source, either peripherally or centrally. Trophic factors were first described as retrograde molecules, but other sources of neurotrophins must also be considered. Our experiments (see below) demonstrate a relevant role of target muscle as a source of neurotrophins for these cells. In fact, muscle expresses all neurotrophins [58,59,62,63,64] and, in particular, BDNF is produced by eye muscles of chick embryos [51]. The alternative ligand for TrkB, neurotrophin NT-4/5, could also be acting from the muscle on extraocular motoneurons. Although the majority of studies have focused on BDNF instead NT-4/5, probably because NT-4/5 was discovered later and both factors use the same receptor, Harandi et al. in 2014 [65] demonstrated the presence of NT-4/5 mRNA in extraocular muscles obtained from SOD1 mice, a mouse model of amyotrophic lateral sclerosis (ALS). In these animals, the expression of NT-4/5 on extraocular motoneurons was low when compared with limb muscles in the control situation, but it increased substantially during the symptomatic period, when the majority of motoneurons, but not extraocular ones, suffer degeneration.

Moreover, neurotrophins that bind to motoneuron Trk receptors might also arise from their afferent inputs [66,67,68,69]. Glial cells may be an additional source of neurotrophins [70], and finally these factors may also act via paracrine or autocrine mechanisms originating from the motoneurons themselves [71,72]. In the case of the oculomotor system, BDNF is present in the vestibular nuclei, one of the major afferent inputs to abducens nucleus [73,74]. Moderate BDNF immunolabeling has also been observed in the abducens nucleus [73,75], suggesting that BDNF could even act as an autocrine or paracrine factor for abducens neurons.

On the other hand, the presence of TrkA receptor in extraocular motoneurons was particularly surprising. Other motoneuronal types express both TrkB and TrkC in the adult [52,58,76,77], but the expression of TrkA in adult non-injured motoneurons seems to be an exclusive feature of extraocular motoneurons [76]. The unique presence or TrkA in adult extraocular motoneurons (as compared to other brainstem or spinal motoneurons) could be causally related to the high resistance of extraocular motoneurons to certain motoneuronal diseases, such as ALS. However, it cannot be discarded that other mechanisms could be implicated in the strong resistance of extraocular motoneurons to neurodegeneration, such as their particular handling of calcium homeostasis. Thus, it has been shown that the transfer of sera from ALS patients to mice increases intracellular calcium and spontaneous neurotransmitter release in spinal but not extraocular motoneuron terminals [78]. Moreover, extraocular motoneurons are enriched in calcium-binding proteins as compared to other motoneuronal types, which might act as buffers of any toxic increase in intracellular calcium levels [79,80].

## 4. The Expression of Trk Receptors Is Differentially Regulated after Axotomy

### 4.1. Changes in Expression of Trk Receptors after Axotomy

**Figure 3:**
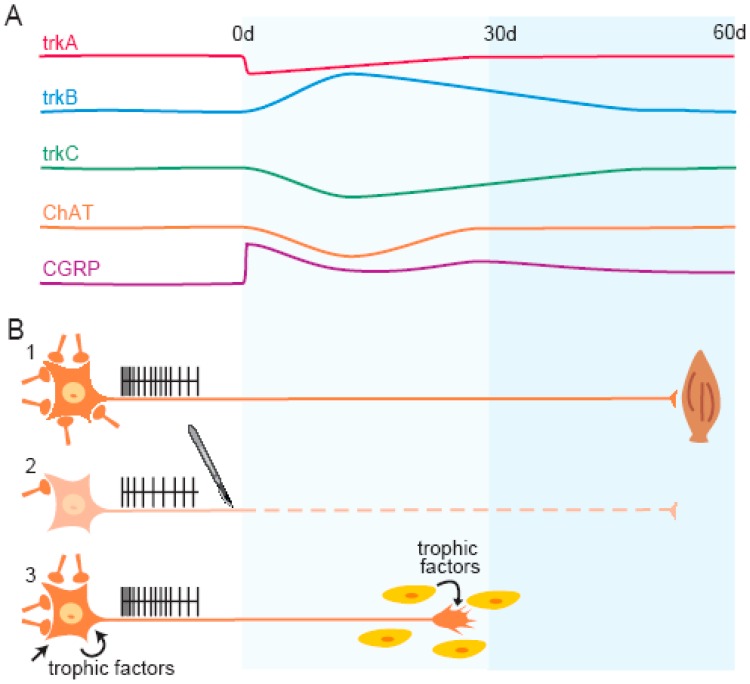
Cartoon indicating alterations in extraocular motoneurons after axotomy: (**A**) time course of changes in the expression of Trk receptor mRNA, ChAT and CGRP (calcitonin gene-related peptide) in extraocular motoneurons after axotomy; d, days; (**B**-**1**) the connected neuron shows a normal phenotype in afferent synaptic activity and firing rate; (**B**-**2**) the axotomized neuron has reduced levels of firing rate and afferent synaptic activity; and (**B**-**3**) the neuron switches from a transmitter to a regenerative phenotype where the autocrine/paracrine and anterograde trophic support gain relative importance with respect to the retrograde trophic support. Appropriate levels of retrograde trophic factors delivered from different sources would restore the values of altered molecules. Modified from [85].

The expression of Trk receptors (TrkA, TrkB and TrkC) in extraocular motoneurons indicates that their associated neurotrophins (NGF, BDNF, NT-4/5 and NT-3) intervene on the normal function of the oculomotor circuitry [49]. As stated above, the main source of neurotrophins for extraocular motoneurons is their target tissue (eye muscles), although they may also arise from afferences or from glial cells. Once neurotrophins are secreted and bound to their corresponding Trk receptors, the ligand-receptor complex is internalized and transported towards the soma, where they initiate the signaling pathway leading to their trophic actions (reviewed in [81]). When motoneurons are deprived of their targets, this trophic supply is interrupted and, as a consequence, a series of alterations on morphological and physiological properties are produced (Figure 3B). In addition, a modification in the expression of numerous molecules involved in survival and regeneration occurs [76,82,83,84].

Extraocular motoneurons can be disconnected from their target by enucleation, a procedure that involves both the injury to the extraocular motor nerves and the removal of target tissues within the eye orbit. In order to investigate the dependence of extraocular motoneurons on neurotrophins we analyzed changes in Trk receptor mRNA levels after enucleation by in situ hybridization [85]. Motoneurons from abducens, trochlear and oculomotor nuclei of the rat are endowed with the machinery to respond to NGF, BDNF and NT-3 since they express basal TrkA, TrkB and TrkC receptors, a common feature with cat extraocular motoneurons. As in the cat, the most abundant Trk receptor in rat extraocular motoneurons is TrkB. The analysis of the changes in TrkA mRNA expression at different times after enucleation revealed a reduction in TrkA mRNA 1–3 days after lesion that recovered to normal values at all the subsequent post-lesion times (Figure 3A).

In contrast, the expression of TrkB mRNA after axotomy was significantly increased in extraocular motoneurons (Figure 3A). The increased expression of TrkB mRNA is indicative of an enhanced responsiveness of these motoneurons to BDNF after axon injury. Indeed, BDNF has been reported to exert important functions for the survival and regeneration of axotomized cranial and spinal motoneurons [1,63,77,86,87]. In contrast, the expression of the specific receptor for NT-3, TrkC, was downregulated in ocular motoneurons at the same post-lesion periods in which TrkB was upregulated (Figure 3A). This opposing effect in the regulation of TrkB and TrkC expression after axotomy has also been reported for other types of motoneuron [60,77,86,88,89,90]. In addition, the infusion of NT-3 is not as efficient as that of BDNF in preventing the death or the downregulation of cholinergic phenotype in injured motoneurons [89,91,92]. Therefore, as it happened with NGF, NT-3 might also not be a key mediator in the survival and regeneration of rat extraocular motoneurons after lesion.

### 4.2. Correlations with Regenerative and Cholinergic Phenotypes

The upregulation of calcitonin gene-related peptide (CGRP), a protein involved in the formation and maintenance of neuromuscular junctions and axonal regeneration, seems to be a common response of cranial motoneurons after axotomy [93]. Extraocular motoneurons did not express CGRP in basal conditions but they did so at short times after axotomy (1–3 days, Figure 3A). This early induction is indicative of a motoneuronal switch to a regenerative state in which new molecules involved in neuronal survival and axonal regeneration can be produced [94,95,96,97,98]. Thirty days after lesion, a new peak in CGRP expression was observed (Figure 3A), which might coincide with the reinnervation of new target tissue, as it has been described for hypoglossal motoneurons [99], or alternatively could be related to an increased availability of trophic support from other sources.

Axotomized extraocular motoneurons also showed a marked decrease in their cholinergic phenotype 15 days after lesion, which was completely restored at longer post-lesion times (Figure 3A). The downregulation of the cholinergic phenotype is a common phenomenon in lesioned motoneurons [60,87,92,100]. The time course evolution of choline acetyltransferase (ChAT) expression after enucleation (an initial decrease by day 15 followed by a recovery; Figure 3A) is coincident with the time course evolution for the increase in TrkB mRNA expression and with that for the decrease in TrkC mRNA expression. These findings point out to a determinant role of BDNF, and not of NT-3, in the restoration of the cholinergic phenotype of the extraocular motoneurons. In fact, BDNF has been shown to attenuate the decrease in ChAT expression in cranial and spinal motoneurons [87,92,101]. After enucleation, reinnervation of axotomized motoneurons with their target is not possible, so the restoration of the cholinergic phenotype likely represents either a truncated regenerative cell program or a response to the increased availability of trophic factors from alternative sources (Figure 3B). This is not the case for other neuronal properties such as firing pattern and synaptic inputs that show a high degree of dependence on target reinnervation for their recovery from the axotomized state [23,24,41,102].

To summarize, expression of TrkB mRNA resulted significantly increased in rat extraocular motoneurons after enucleation whereas expression of TrkA and TrkC mRNAs were decreased. This indicates that, among neurotrophins, BDNF might have a main role in the restoration of the cholinergic phenotype as well as in the survival and regeneration of the axotomized motoneurons. We can conclude that extraocular motoneurons differentially modify their requirement of neurotrophins as a consequence of their target removal.

## 5. Neurotrophins Rescue Extraocular Motoneurons from Axotomy-Induced Cell Death during Postnatal Development

Dependence on neurotrophic supply for motoneuronal survival varies throughout maturation. During development, motoneurons are strongly dependent on neurotrophic support arriving from the target muscle for survival, and any interruption of this retrograde flow leads to a massive loss of motoneurons. As motoneurons mature, they lose their dependence on neurotrophic factors for survival, but when they are disconnected from their target, they experiment several alterations in electrophysiological properties and protein expression that can be recovered if they are provided with a new target [102,103,104], but also by the exogenous administration of different neurotrophins [105,106,107,108].

### 5.1. Survival of Extraocular Motoneurons after Postnatal Axotomy and Neurotrophin Administration

In the same way, extraocular motoneurons depend on their target muscles for survival during postnatal development, but this dependence is lost with maturation [38]. Previous studies had explored the administration of diverse neurotrophins as a method to reduce axotomy-induced cell death in neonates, resulting in diverse degrees of survival [63,109,110,111,112,113]. We demonstrated that extraocular muscles of neonatal rats express the neurotrophins NGF, BDNF and NT-3 [114], and also that motoneurons of the oculomotor system contain the TrkA, TrkB and TrkC receptors [49,85], indicating that neurotrophins delivered from the target likely play a relevant role in extraocular motoneurons. In order to uncover the degree of dependence of extraocular motoneurons on different neurotrophins in the period in which they are more vulnerable to the deprivation of neurotrophic supply, newborn rats were monocularly enucleated as a procedure to axotomize extraocular motoneurons, and different neurotrophins were applied to the orbit immediately after lesion [114].

**Figure 4:**
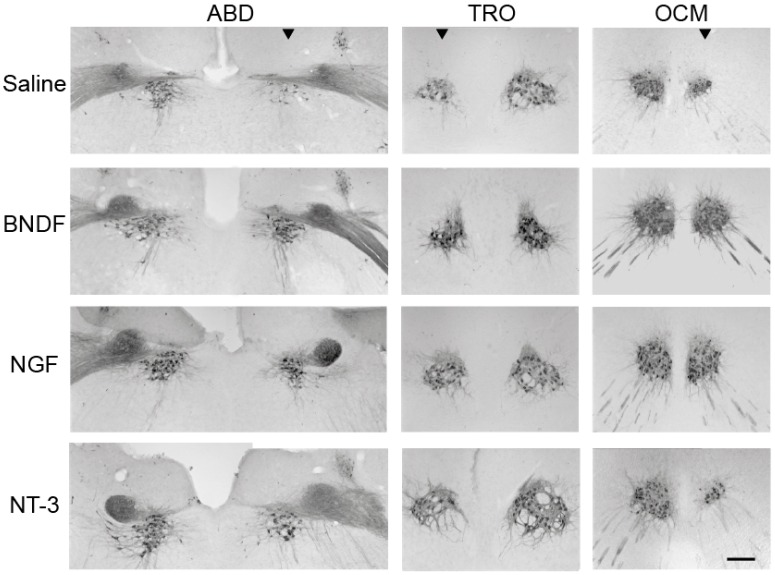
ChAT immunoreactivity in extraocular motoneurons 10 days after right eye enucleation at P0 and different neurotrophic treatments from the orbit via a Gelfoam implant. Note the remarkable reduction in cell death in the different axotomized extraocular motor nuclei, as compared with the saline treatment, after the administration of BDNF, NGF or NT-3. BDNF and NGF, but not NT-3, also prevented the downregulation in ChAT in the lesioned motoneurons of the abducens (ABD), trochlear (TRO) and oculomotor (OCM) nuclei. Arrowheads indicate the affected side for each nucleus after the unilateral enucleation. Calibration bar: 200 µm. Modified from [114].

Neonatal enucleation in P0 resulted in a massive death of approximately 65% of extraocular motoneurons by 10 days after lesion [114]. The exogenous administration of diverse neurotrophic factors at the moment of the axotomy was performed by the application of Gelfoam implants soaked in diverse neurotrophins (NGF, BDNF or NT-3) into the enucleated ocular orbit. By 10 days post-lesion, all treatments significantly increased motoneuron survival following axotomy, as can be observed in Figure 4, when comparing with saline treatment. Motoneuronal survival differed between treatments, with NGF as the best factor preventing extraocular motoneuron death, followed by BDNF, being NT-3 the neurotrophin showing the lowest effect on survival (88%, 75% and 68% of survival obtained after NGF, BDNF and NT-3 treatments, respectively).

The large percentage of survival obtained after the administration of NGF was surprising, as this result contrasts with previous findings showing that NGF is unable to rescue immature spinal or facial motoneurons in either lesioned neonatal rats or cultures [111,112,115]. It is important to highlight that extraocular motoneurons are peculiar because they express TrkA in control conditions, as explained above [49,85]. By contrast, TrkA expression is not seen in other adult brainstem or spinal motoneurons [58,63], suggesting an important functional role of NGF in the regulation of survival and physiological properties of extraocular motoneurons. Interestingly, it is well known that extraocular motoneurons are much less vulnerable than other brainstem and spinal motoneurons to degenerating motoneuron diseases such as ALS [79,116,117,118], and spinal motoneurons that survive ALS in human patients have shown an increased TrkA expression, suggesting that NGF may provide trophic support to surviving motoneurons in this disease [119]. Thus, the unique responsiveness of extraocular motoneurons to NGF could be contributing to their longer survival detected in ALS.

We also found that the administration of BDNF to axotomized neonatal extraocular motoneurons was very effective in rescuing these cells from cell death. The effect of BDNF on survival of lesioned motoneurons has been studied in other neonatal models, resulting in the prevention of the axotomy-induced cell death of facial and spinal motoneurons [63,110,111,113]. In addition, adenoviral gene therapy using recombinant adenovirus vectors encoding BDNF cDNAs has shown the ability to promote the survival of spinal, facial or ambiguous nucleus motoneurons after avulsion in adult rats [120,121].

In agreement with our data in extraocular motoneurons [114], NT-3 applied to severed motoneurons in newborn rats also exhibit some ability to rescue motoneurons from cell death, but less efficiently than BDNF, both in spinal [122] and facial [111] motoneurons. In the adult rat, the administration of NT-3 exerts no protective effect on cell death in hypoglossal motoneurons after nerve transection [92] or in corticospinal neurons after central lesion [123].

### 5.2. Maintenance of the Cholinergic Phenotype in Extraocular Motoneurons by Neurotrophins after Axotomy

As mentioned previously, axotomy produces a transient but reversible loss of the cholinergic phenotype in extraocular motoneurons of the neonatal and adult rat [124]. We found that BDNF or NGF administered at the same time of axotomy prevent the downregulation of ChAT expression found 10 days after lesion in extraocular motoneurons. Again, NT-3 showed the least beneficial effect on ChAT downregulation among all the different neurotrophins used (95%, 77% and 42% of ChAT-positive motoneurons 10 days after NGF, BDNF and NT-3 administration, respectively). Thus, NT-3 was able to rescue axotomized motoneurons from cell death, but was not effective enough to prevent the loss of the cholinergic phenotype observed in these motoneurons by 10 days after lesion.

The downregulation in ChAT is a common phenomenon described not only in the oculomotor system, but also in hypoglossal, facial and spinal motoneurons [92,100,101,125,126]. In injured hypoglossal, facial and spinal motoneurons, the administration of neurotrophic factors has also been proven as an effective method to prevent the loss of the cholinergic phenotype. In line with our results, BDNF has been shown to exert a protective effect against the loss of ChAT, and NT-3 was ineffective in preventing the decrease in ChAT after axotomy in other types of motoneuron [60,92]. The high efficacy of BDNF could be attributed to the upregulation in TrkB receptors observed shortly after lesion in extraocular (described above, [85]), and in facial motoneurons [60], whereas TrkC receptors experiment a downregulation in axotomized motoneurons. However, NGF failed to sustain the cholinergic phenotype in hypoglossal and facial motoneurons after axotomy, probably due to the lack of TrkA receptors in these motoneurons [60,92,100], in opposition to the expression of TrkA by extraocular motoneurons [49,85].

### 5.3. Long-Lasting Effects of Neurotrophic Factors in Extraocular Motoneurons

In neonatal rats, the effect of the administration of neurotrophins on extraocular motoneuron survival at a longer post-lesion time revealed that the prevention of motoneuronal death observed 10 days after axotomy was also present by 30 days post-lesion [114]. These results indicate that the enhanced survival induced by these neurotrophins was not transient, but rather extended throughout a long period.

Our results have shown that motoneuronal death in neonatal animals after axotomy occurred mainly in the first 10 days after lesion, consistent with observations made by other authors [109]. In addition, all the neurotrophic factors applied produced the long-term survival of axotomized extraocular motoneurons, at least up to 30 days. Similar results were obtained in neonatal rats after sciatic nerve axotomy, where both BDNF and NT-3 were able to support the long-term survival of dorsal root ganglion neurons [127]. BDNF produces also long-lasting survival of other neuronal pools, like corticospinal neurons [123], whereas NT-3 fails to protect upper motoneurons in the same model. However, neurotrophins do not show long-lasting neuroprotective effects in all types of motoneuron. In axotomized neonatal spinal motoneurons, the administration of a variety of neurotrophic factors enhance motoneuronal survival at short time post-lesion, but their effects are only transitory as a great number of motoneurons dies at longer survival time [112,128].

In conclusion, our results showed that the administration of the neurotrophins NGF, BDNF or NT-3 were able to rescue extraocular motoneurons from axotomy-induced cell death after enucleation performed in newborn rats, showing long-lasting neuroprotective actions. NGF was the most effective neurotrophin in preventing death of axotomized extraocular motoneurons, followed by BDNF, while NT-3 showed the least effect as a neuroprotective factor. In addition, NGF and BDNF prevented the downregulation in ChAT expression induced by axotomy, though NT-3 failed to maintain the cholinergic phenotype of these motoneurons. It is remarkable the high efficacy of NGF as a neuroprotective factor for injured extraocular motoneurons in contrast with the results obtained in other motoneuronal types.

## 6. BDNF and NT-3 Exert Complementary Actions on the Discharge Activity of Extraocular Motoneurons

As stated above, axotomy induces a plethora of changes in the damaged motoneurons, including modifications at both the structural and functional level [129,130], which remain altered as motoneurons are disconnected from their target, but may revert when motoneurons are able to reinnervate their target [23,131,132,133,134]. All these evidence point to target-derived molecules as the effectors of phenotype maintenance.

In the case of the oculomotor system, the fact that, first, extraocular motoneurons express the three Trk receptors, and second, their expression is modulated in response to target depletion, let us argue for a principal role of neurotrophins in the maintenance of oculomotor circuitry and the establishment of the adequate firing signals that govern all types of eye movements. If this hypothesis is correct, then not only neurotrophin removal by target depletion would diminish those eye-related firing signals, but also the exogenous administration of neurotrophins would be enough to restore normal firing after the interruption of the normal muscle-motoneuron retrograde trophic signaling.

To test this prediction, different neurotrophins were administered to axotomized abducens motoneurons. In a first set of experiments, we injected either BDNF, or NT-3, or a combination of both neurotrophins, to the distal stumps of sectioned axons, following two different experimental paradigms: in the first one, we administered the neurotrophin immediately following axotomy, to test the maintenance of motoneuron properties; in the second one, we first axotomized motoneurons and then checked whether neurotrophins could revert the effects of the lesion. To prevent lesioned motoneurons to establish contacts with new targets, we developed an administration device for chronic neurotrophin delivery, which allowed controlling the extracellular environment that bathes the axonal stumps [135]. In a second set of experiments, we injected NGF following the same paradigms explained above. We will discuss NGF results in the following section.

**Figure 5:**
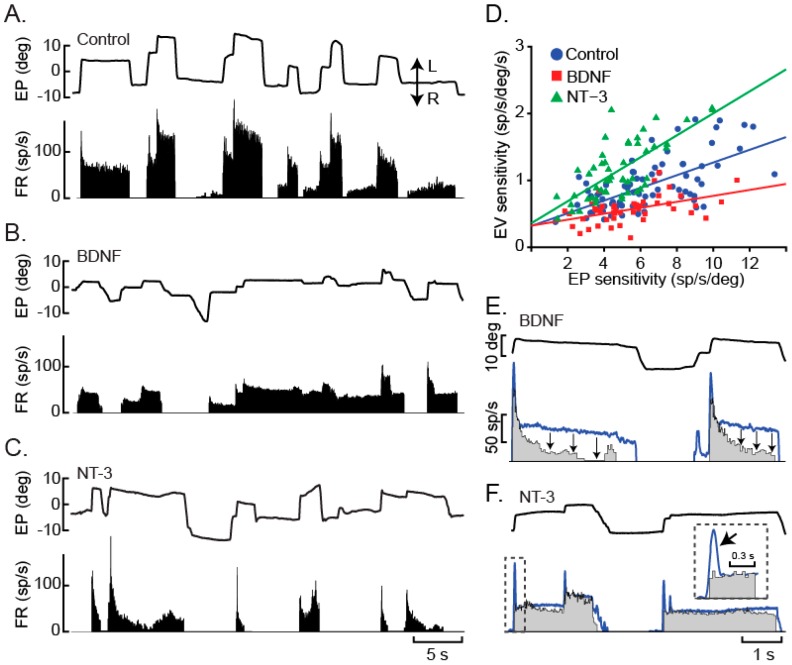
Complementary firing patterns of BDNF- and NT-3-treated motoneurons: (**A**) The firing rate (FR, in spikes/s; sp/s) of control motoneurons shows burst for on-directed saccades and a tonic activity during eye fixations; (**B**) treatment with BDNF for eight days immediately after axotomy reduced bursts in response to saccades; (**C**) same as (**B**) but for a seven-day NT-3-treated cell displaying large bursts for saccades but reduced tonic firing for eye fixations; (**D**) relationship between neuronal eye position (EP, in degrees; deg) and eye velocity (EV) sensitivities obtained by linear regression analysis for 75 control (**blue**), 45 BDNF-treated (**red**) and 51 NT-3-treated (**green**) motoneurons showing that BDNF recovered the tonic (EP), whereas NT-3 restores the phasic (EV) component of discharge activity; and (**E**) response of a motoneuron (**grey** firing rate) NT-3-treated for 14 days immediately after axotomy. The blue line represents the firing rate simulation expected for the actual eye movement. Arrows point to the decay in firing rate during fixations; (**F**) Response of a BDNF-treated motoneuron (**grey**) for 24 days demonstrating a tonic mode of firing. The blue line as in (**E**) represents the simulated firing expected for the actual eye movements. The arrow points to the reduced bursts during saccades that are characteristic of BDNF-treated cells that, however, exhibit normal tonic firing. Taken from [105].

We found that BDNF and NT-3 could not only prevent, but also revert, the effects of axotomy, although only partially. Thus, none of them, when administered alone, was able to maintain or restore completely motoneuron synaptic inputs, and thus, firing pattern. Instead, the administration of either BDNF or NT-3 produced complementary effects on the maintenance/recovery of motoneuron discharge activity, and, indeed, the combined administration of both neurotrophins led to a complete functional and morphological restoration of normal motoneuron characteristics [105] (see Figure 5). Neurotrophins exert their function through Trk signaling, since the simultaneous administrations of neurotrophins and K252a, a selective inhibitor in vivo and in vitro of Trk action [136,137,138], abolished neurotrophin-mediated effects.

**Figure 6:**
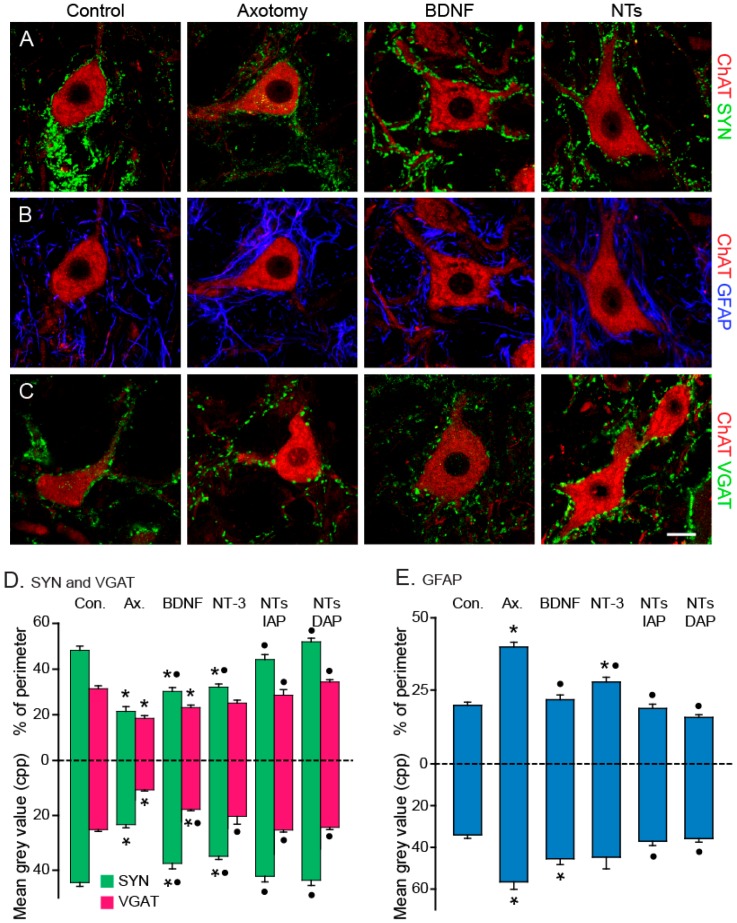
Neurotrophin involvement in prevention of synaptic stripping: (**A**) Confocal high-magnification images of motoneurons in control, axotomy, and after treatment with BDNF or with the mixture of BDNF+NT-3 (NTs), illustrating the synaptophysin (SYN) innervation in green and the motoneuronal cell body in red (ChAT). All images were obtained from animals after 15 days of axotomy alone or after treatment with neurotrophins in an immediate administration protocol (IAP); (**B**) Same treatments as in (A), but for GFAP coverage; (**C**) Confocal images of VGAT labeling in ChAT-identified motoneurons. Calibrations bar: 10 µm for (**A**–**C**); (**D**) Changes in SYN-immunoreactive (IR; **green**) and VGAR-IR (Simbols to be erased) boutons in the abducens nucleus after axotomy (Ax.) and different treatments (Con., control). Up-directed bars represent the percent of covered perimeter of the motoneurons in control, axotomy, single neurotrophin treatment (BDNF or NT-3) and double neurotrophin treatment for either IAP or delayed administration protocol (DAP). Down-directed bars correspond to measurements in the neuropil. Bars represent mean ± SEM for 15–46 motoneuronal profiles in each group and 26 to 50 measurements in the neuropil; (**E**) Same as in (**D**) but for measurements of GFAP-IR profiles around somata (up directed bars) or in the neuropil (down-directed bars). Asterisks indicate significant differences with control, whereas dots indicate significant differences with axotomy. Two way ANOVA, Holm–Sidak method for pairwise multiple comparisons (*p* < 0.01). Modified from [105].

Control abducens motoneurons exhibit a typical tonic-phasic firing pattern, as recorded in vivo under alert conditions (Figure 5A). Axotomy induces a reduction in both discharge components (i.e., tonic and phasic [105]). A striking result was obtained after the administration of BDNF or NT-3 to axotomized motoneurons, since their effects were complementary and additive. In particular, BDNF prevented/restored the decrease in tonic firing signals related to eye position, whereas NT-3 supported the phasic component (Figure 5B–F). For instance, axotomy induces a diminution in burst firing during eye movements directed towards the ipsilateral visual hemifield. This reduction was avoided/reverted after NT-3 administration. These results pointed to separate synaptotrophic actions of BDNF and NT-3 on afferent neurons to abducens motoneurons. According to neurophysiological data, animals treated with the cocktail of these two neurotrophins exhibited a recovery of both (the tonic and the phasic) components of the discharge activity as well as a normal pattern of afferent inputs. Thus, using immunostaining against synaptophysin (as a marker of presynaptic boutons), glial fibrillary acidic protein (GFAP, as a marker of astrocytes) and vesicular transporter of GABA and glycine (VGAT, as a marker of inhibitory synaptic boutons), we observed a significant decrease in both synaptophysin- and VGAT-labeled boutons (Figure 6A,C) and a significant increase in astrocytic reaction following axotomy (Figure 6A,B), as confirmed in the quantitative analysis (Figure 6D,E). Only the treatment of BDNF along with NT-3 (NTs in Figure 6) reestablished a normal synaptic complement and astrocytic density as compared to control (Figure 6D,E), whereas the treatment with BDNF of NT-3 alone produced intermediate values between control and axotomized motoneurons (Figure 6D,E).

Neurotrophin expression is strongly affected by modification in synaptic activity, and vice versa, changes in neurotrophin availability can modify synaptic transmission [139,140]. In fact, it has been shown that BDNF and NT-3 strongly regulate the synaptic composition and the formation of pre- and post-synaptic elements in the adult brain [11,141] and in vitro [142,143]. In line with our results obtained in the oculomotor system, BDNF and NT-3 have also opposing roles in the electrophysiological properties of the spiral ganglion neurons [144,145] and in the growth of dendrites in layer 4 pyramidal neurons [142].

In abducens motoneurons, NT-3 produced specifically the recovery of the phasic pattern of firing. Phasic firing is coded through the combined action of pontine excitatory and inhibitory reticular neurons [30,32,146], that command, respectively, bursts of action potentials during on-directed rapid eye movements and pauses of activity during movements in the opposite direction [28]. According to these results, NT-3 is likely to display trophic effects over both excitatory and inhibitory synaptic terminals of reticular afferent neurons (EBN and IBN, see Figure 1) contacting on the motoneurons. In fact, exogenous NT-3 treatment resulted in increased eye velocity sensitivity in motoneurons, even when compared with control. Similarly, Ia synapses over axotomized spinal motoneurons are also affected by NT-3 [147,148,149,150].

On the other hand, BDNF mediates tonic firing of abducens motoneurons as well as the maintenance of related synaptic inputs. The tonic component is codified by a velocity-to-position integrator [151] located at the prepositus hypoglossi nucleus [34,152] (see Figure 1), which innervate bilaterally abducens motoneurons, providing proportionally increasing excitation as eye fixations are located in more abducting positions, as well as inhibition for fixations in the opposite direction. Although BDNF increases cell excitability by modulating potassium and sodium currents [153,154], the effects of this molecule on abducens motoneuron firing pattern are more probably related to a role in prepositus afferent synapse modulation. In agreement with this hypothesis, BDNF also regulates the synaptic composition of spinal motoneurons [11].

Besides reticular and prepositus afferences, abducens motoneurons are also innervated by the medial vestibular nucleus, which provides reciprocal, excitatory-contralateral/inhibitory-ipsilateral input to the abducens motoneurons (see Figure 1). Thus, vestibular neurons convey an integrated position-velocity signal that guide eye movements during the vestibulo-ocular reflex, in collaboration with prepositus neurons [33,155,156]. Excitatory vestibular synaptic potentials were recovered in axotomized abducens motoneurons following BDNF treatment, suggesting that synaptic inputs from contralateral vestibular neurons could depend on the trophic action of BDNF. On the other hand, NT-3 delivery restored inhibitory vestibular synaptic potentials, indicating a relevant role of NT-3 on the maintenance of synapses between ipsilateral vestibular neurons and abducens motoneurons.

Altogether, it can be concluded that BDNF and NT-3 exert their influence on different synaptic inputs to abducens motoneurons and therefore, the administration of only one of these molecules re-establishes partially the discharge activity of axotomized motoneurons. Complementary and additive effects of BDNF and NT-3 are revealed when both factors are administered simultaneously, leading to the complete recovery of axotomized motoneuronal firing.

## 7. NGF Recovers above Control the Discharge Activity of Axotomized Motoneurons: Differential Role of TrkA and p75 Receptors

Skeletal muscle expresses NGF exclusively during development [64]. In the adult, its expression is downregulated [157,158], and it is only under certain circumstances, like muscle repair or pathology, which this neurotrophin is expressed again [159]. Accordingly, cranial and spinal motoneurons express TrkA only transiently during development, and the later also after lesion or in response to some musculoskeletal pathologies [160,161]. Intriguingly, healthy adult extraocular motoneurons normally express the NGF high and low affinity receptors [49,106], suggesting a possible role for this neurotrophin in the maintenance of oculomotor circuitry and their motoneuron physiology. To test this hypothesis, NGF trophic support was altered on abducens motoneurons by either axotomy or applying chronically exogenous NGF to the stump of the sectioned nerve through the above mentioned substance administration device [135]. To further study if the effects of the exogenous NGF were derived from its binding to its high (TrkA) or low affinity receptor (p75^NTR^), we combined the neurotrophin with two different drugs: K252a, a selective inhibitor in vivo and in vitro of Trk action [136,137,138], or REX, a polyclonal antibody against the rat extracellular domain of p75^NTR^ [162].

Although it has been previously described that NGF, acting through its low affinity receptor, p75^NTR^, can produce cell death and massive pruning in developing retinal cells and other neurons lacking TrkA [163,164], the role of p75^NTR^ is not always detrimental for neurons. This receptor plays an important role increasing TrkA efficiency to enhance cell survival during development and in the adult [164,165,166]. In fact, the coexistence of these two receptors can facilitate Trk signaling, selective binding of neurotrophins to their different Trk receptors, and uptake of neurotrophins for retrograde transport [167,168,169]. Consequently, the presence of both receptors on abducens motoneurons and the modulation of their expression after lesion might provide beneficial effects to these neurons after lesion and in musculoskeletal disease [49].

**Figure 7:**
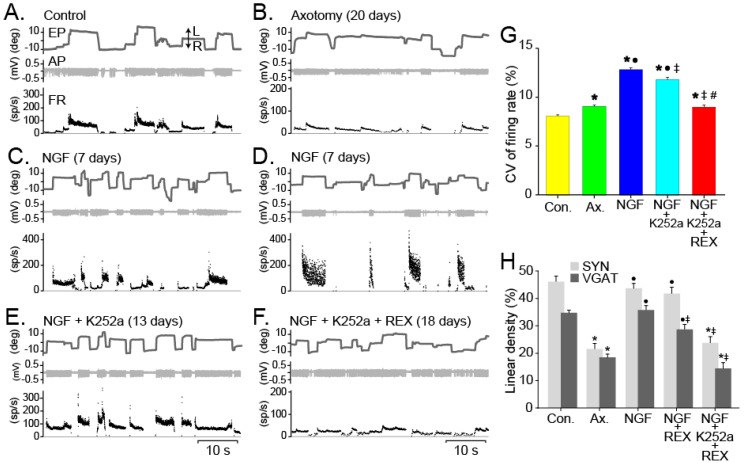
Effects of NGF and NGF receptor blockers on the discharge pattern of axotomized abducens motoneurons: (**A**) Firing rate (FR) profile of a control abducens motoneuron recorded during spontaneous eye movements (EP, eye position). The single-unit discharge of action potentials (AP) is shown in the middle trace. L and R are left and right directions of movement, respectively; (**B**) Same as in (**A**), but for a motoneuron recorded 20 days after axotomy. Note reduced firing during both saccades and spontaneous fixations; (**C**,**D**) NGF treatment causes recovery of the burst-tonic firing pattern; however, the discharge variability increased during eye fixations; (**E**) Motoneurons treated with K252a still showed some of the effects produced by NGF, indicating that part of the effects of NGF could be mediated also via the p75^NTR^; (**F**) Motoneurons treated with K252a and the p75^NTR^ blocker REX demonstrated an axotomy-like firing profile although they were firing continuously; (**G**) Comparison of the coefficient of variation (CV) of firing rate in the interval 30–80 sp/s. Note that NGF treatment dramatically increased the firing variability, while blockade of TrkA receptors partially decreased variability, and blockade of both p75^NTR^ and TrkA receptors restored firing variability to axotomy levels (ANOVA test, Holm–Sidak method for pairwise multiple comparisons, *p* < 0.05). Symbols indicate significant differences with respect to: *, control; •, axotomy; ‡, NGF treatment; and #, NGF+K252a treatment; (**H**) Linear density of synaptophysin-IR (SYN) and VGAT-IR boutons over the somatic perimeter of motoneurons changes after axotomy and the different treatments. Bars represent mean ± SEM for 17–38 motoneuronal profiles in each group. Asterisks indicate differences with control, dots with axotomy, and double crosses with the NGF group. Modified from [106].

Administration of NGF to the nerve stump of abducens motoneurons immediately after the lesion, not only prevented the decay in eye position and velocity sensitivities caused by axotomy (Figure 7A,B [23,105]), but also produced a significant rapid increase in their sensitivities to eye related parameters (Figure 7C,D) that were maintained higher than those of control motoneurons throughout the course of the experiment [106]. The short- and long-term effects of neurotrophins on synaptic transmission and plasticity have been well documented (reviewed in [170,171]). NGF produces increases of nicotinic transmission in parasympathetic motoneurons that are dependent on TrkA [172]. In the mouse medial septum/diagonal band of Broca, this neurotrophin directly enhances activity in cholinergic neurons. In some of these cells, NGF not only increases acetylcholine release but also glutamate transmission and the expression of acetylcholine-related enzymes, such as ChAT, acetylcholinesterase, and vesicular acetylcholine transporter [173,174,175,176], resulting in an overall increase of muscarinic tone. In vitro studies show that short-term actions of NGF were dependent on neuron activity and act through a TrkA dependent pathway, however, they do not rule out the possibility that some of the neuromodulatory effects of NGF are produced by the activation of p75^NTR^ [177]. In agreement with this, Huh and collaborators [175] showed that long-term neuromodulatory effects of NGF on cholinergic neurons of the same area were p75^NTR^ dependent, as it is the NGF dependent glutamate release from cerebellar neurons [178] or dopamine release from mesencephalic neurons [179]. In our experiments, neuronal sensitivities were partly dependent on both NGF receptors, because K252a treatment decreased eye-related sensitivities to levels similar to control motoneurons (Figure 7E), whereas dual receptor blockade produced a further decay to axotomy levels (Figure 7F). This result indicates that, as has been shown by others, both receptors act in a cooperative fashion [167]. In this sense, NGF acting through the p75^NTR^ alone was sufficient to restore the firing sensitivity of the motoneuron, but when acted together with the TrkA receptor, the effects were additive, resulting in sensitivities that rose above control.

Recruitment threshold, calculated as the abscissa intercept of the rate-position plot, changed after NGF treatment [106]. This parameter indicates the eye position at which the motoneuron first enters into activity. Studies in spinal motoneurons indicate that recruitment threshold varies with the synaptic input organization and activity patterns of afferent neurons [180,181,182]. Therefore, changes in this parameter might indicate changes in the organization or balance between the inhibitory vs. excitatory inputs that the motoneuron receives. Thus, if this ratio is altered, there are basal activity changes. This was demonstrated on abducens motoneurons by injection of tetanus toxin, a neurotoxin that produces transynaptic deafferentation, on the lateral rectus muscle [183,184]. Thus, low doses of tetanus toxin produce disinhibition and, therefore, a low inhibition-to-excitation ratio that decreases recruitment threshold. Although axotomy did not affect recruitment threshold of abducens motoneurons, when these cells were treated with NGF, not only they did show higher sensitivities to eye movements, but they also increased recruitment threshold. The later increase was maintained even when the TrkA receptor was blocked, indicating that the shift in this variable was due to NGF acting through its low affinity receptor p75^NTR^. Furthermore, when both types of NGF receptors were blocked, the recruitment threshold returned to axotomized values. Since this variable is related to the signals that the cells receive, NGF-related high recruitment threshold registered on axotomized abducens motoneurons might be due to changes in the balance between inhibitory vs. excitatory afferences. In fact, these cells showed ample inhibition, as demonstrated by longer firing pauses during spontaneous eye movements (Figure 7C,D) and high somatic coverage with VGAT (Figure 7H). In line with our results, NGF acting through p75^NTR^ has been shown to increase the ratio of inhibition to excitation on hippocampal neurons [185]. In parallel with this change, NGF-treated cells also demonstrated more excitation, as shown by larger eye position sensitivity values. These larger synaptic drives might be possible by greater synaptic efficacy, since analysis of the synaptic coverage of these cells showed no vacant synaptic space [172]. Thus, as it has been seen in other circuits [186], changes in the amount of inhibition seemed to homeostatically compensate for changes in the excitatory drive. To the contrary, cells treated with both NGF receptor blockers, showed a low excitatory drive, they fired in a continuous, low modulation mode (Figure 7F) and presented low eye position sensitivity in the off direction (an index of the inhibition they received), as if they were disinhibited. In addition, these cells exhibit lower values of VGAT somatic coverage than expected (Figure 7H), suggesting that excitatory changes also promote adjustments in the inhibitory drive [187].

Furthermore, treatment with NGF to axotomized abducens motoneurons, also impeded the synaptic stripping of afferent innervation on these neurons [188], which is one of the main anatomical effects of axotomy [23]. Deafferentation is caused by target depletion and not by lesion itself, since if neurons reinnervate any other target this effect disappears [41]. Somatic synaptic coverage on axotomyzed abducens motoneurons only decreased when NGF was administered together with both receptor blockers, but not when only p75^NTR^ was blocked (Figure 7H). This result suggests that the prevention of loss of synaptic inputs to injured motoneurons was mainly mediated by the TrkA receptor. In consonance with this, NGF acting through its high affinity receptor, is known to regulate different aspects of synaptic transmission and efficacy [176,189], and to promote long term changes in synaptic organization [172].

Finally, treatment with this neurotrophin produced changes in the variability of the firing discharge of abducens motoneurons. To study these changes we analyzed and compared the coefficient of variation (i.e., the percentage ratio of the SD to the mean) in the frequency range where this variable was relatively stable, that is, between 30 and 80 sp/s (Figure 7G). While axotomy induced a slightly (but significant) increase in firing variability of abducens motoneurons, treatment with NGF evoked a much larger rise that barely decreased when the TrkA receptor was blocked. These results suggest that the effects of NGF on the firing variability of motoneurons are mainly controlled through activation of p75^NTR^. In fact, this parameter returned back to axotomy levels only when both receptors were blocked. In accordance with our results, activation of the p75^NTR^ on Purkinje cells regulates the function of small conductance Ca^2+^-activated K^+^ (SK) channel, and thus is involved in the maintenance of the firing regularity of these cells in the adult cerebellum [190]. Moreover, NGF has been shown to regulate excitability and firing regularity in other neuronal types through several ionic currents [191,192]. One of them is the M-current, likely present in oculomotor motoneurons, as seen with carbachol-induced depolarizations [193]. In primary sensory neurons, NGF acting through p75^NTR^ [191,194] or TrkA [195,196,197] induces pain sensitization due to changes of expression and kinematics of different receptors and ion channels that contribute to the immediate hyperexcitability of the nociceptor after inflammation (reviewed in [198]). Since irregularity of firing in neurons can arise from the external stimuli the cell receives [199,200,201,202] or from intrinsic changes leading to alterations in the kinetics of ion channels [203,204], further studies are needed to define the mechanism by which NGF alters firing variability of abducens motoneurons.

In summary, extraocular motoneurons are peculiar in the sense that they respond to NGF, which is not the case for other motoneuronal types. The administration of NGF to axotomized abducens motoneurons not only recovers the tonic and the phasic components of the discharge, but even increases both components above control values. Using selective blockers, we have demonstrated different actions mediated by NGF depending on the receptor activated, i.e., TrkA or p75^NTR^. Thus, while TrkA signaling supports synaptic afferences and regulates the eye-related burst and tonic signals of abducens motoneurons, p75^NTR^ activation affects the recruitment threshold and the firing regularity of these motoneurons.

## 8. A Hypothesis Linking Neurotrophic Delivery with Motoneuronal Types and Their Afferences

We have shown that target-derived dependencies extend from the mere survival during development and early postnatal life to the regulation of phenotypical properties in the adult state. Neurotrophins seem to play an essential role during both phases in the life of a motoneuron. In this section we might like to speculate that access to different sources and combinations of trophic factors could lead to variations in the expression of physiological (i.e., synaptic and firing) properties of motoneurons.

**Figure 8:**
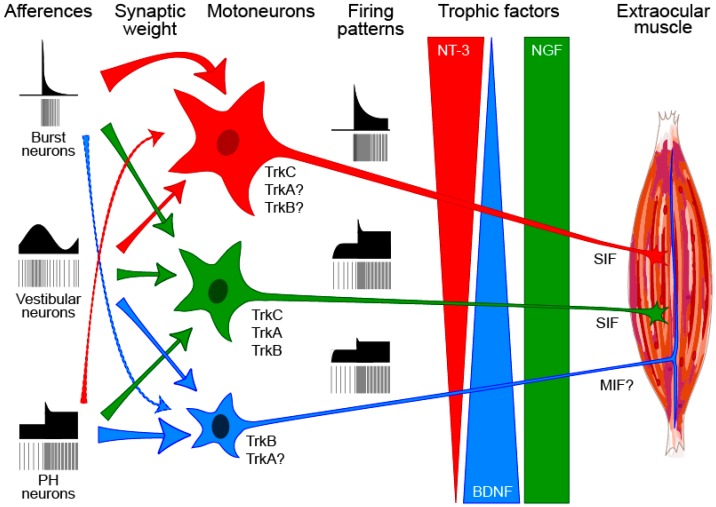
Postulated trophic classification of extraocular motoneurons based on their accessibility to different sources of neurotrophins. Different neurotrophins produced by multiply-innervated (MIF) and singly-innervated (SIF) muscle fibers could be secreted and accessible to motoneurons. Complementary gradients of BDNF and NT-3 or homogeneous supply of NGF by the muscle will reach in different proportions to the soma of motoneurons via their complement of Trk receptors. It is postulated that some motoneurons will not be endowed with all three receptors. Trk signaling will lead to a synaptotrophic scaling of the three main afferents to abducens motoneurons generating thus the three semantically distinct groups of motoneurons that could both differ in size and signaling properties. From top to bottom, phasic (**red**), tonic-phasic (**green**) and tonic (**blue**) proposed motoneuronal types.

Although the division of labor into different classes of motoneuron and muscle fiber is well established from the early work in spinal cord [205,206], the oculomotor motoneurons have remained resilient to classification into different types for decades. The tonic-phasic firing pattern is homogeneous through the entire population of abducens motoneurons [37,105], and motoneurons are part of a continuum rather than being clustered in different types [207]. It should also be pointed out that both, in experimental and modeling grounds, the classification of spinal motor units begins to be contemplated more like a continuum along the different physiological properties tested, and the grouping into types appears as a semantic classification [208]. Nonetheless, at least two types of motoneurons have been proposed recently on the basis of retrograde and transneuronal tracing (i.e., twitch large cells and small, non-twitch cells, peripherally located in the nucleus [209,210]; see Figure 8).

Our past work summarized in Section 6 and Section 7 opens the possibility for the existence of different motoneuronal types (tonic, phasic and mixtures of both) according to their responsiveness to different neurotrophins arising from the target. Perhaps the differential availability could be directed by the production of different cellular components of the muscle on one hand, and in the other for the distinct composition of Trk receptors on the motoneuron. Taking into account that around 80% of extraocular motoneurons coexpress TrkB and TrkC [49], it could be concluded that the vast majority of these motoneurons use the combination of BDNF and NT-3 obtained from the target to maintain their normal synaptic input and firing pattern. However, approximately 20% of cells might depend on only one or two neurotrophins. This would indicate that some control motoneurons might be purely tonic, others purely phasic and the vast majority tonic-phasic (Figure 8).

We have shown that BDNF induces tonic firing in motoneurons via TrkB receptors, whereas NT-3 induces phasic firing through TrkC receptors [105]. We postulate that retrograde influences may change the gradient of firing patterns, from tonic to phasic, as recently described for sympathetic neurons [192] by changing the synaptic weight of the afferences to motoneurons. Changes in the trophic support could explain phenotypic changes in spinal motoneurons affected of ALS that change from a BDNF/NT-3 signaling to NGF [119]. Other changes in neurotrophin signaling have been observed in other diseases such as diabetic neuropathy [211], cardiac arrests [212] and Parkinson’s disease [213]. Therefore, changes in the expression of target-derived factors and the signaling of the innervating cells should be part of the investigations on the etiology of many diseases.

## 9. Conclusions

We have shown that target cells are powerful regulators of the phenotypical properties of innervating cells not only through early stages of development but also later throughout life in the adult state. Target disconnection by axotomy leads to a myriad of alterations that may range from cell death to physiological, metabolic and structural perturbations. The administration of neurotrophins to axotomized neurons can restore, at least in part, some of the axotomy-induced alterations, although each neurotrophin recovers different aspects of cell physiology. Moreover, neurotrophins show complementary and additive effects and may mediate different actions through Trk and p75^NTR^ receptors, whose expression is modulated in response to injury. In this review, we have focused on the oculomotor system, in which neurotrophins act as key regulators of cell survival and neuron function and structure, but we also have extended and discussed our results to those found in other systems.
